# Modulation of low-dose ozone and LPS exposed acute mouse lung inflammation by IF1 mediated ATP hydrolysis inhibitor, BTB06584

**DOI:** 10.3389/fimmu.2023.1126574

**Published:** 2023-03-13

**Authors:** Pahul Singh, Gurpreet Kaur Aulakh

**Affiliations:** Department of Small Animal Clinical Sciences, Western College of Veterinary Medicine, University of Saskatchewan, Saskatoon, SK, Canada

**Keywords:** ozone, LPS, acute lung inflammation, IF1, propranolol, BTB06584, CD61, dexamethasone

## Abstract

Ozone and bacterial lipopolysaccharide (LPS) are common air pollutants that are related to high hospital admissions due to airway hyperreactivity and increased susceptibility to infections, especially in children, older population and individuals with underlying conditions. We modeled acute lung inflammation (ALI) by exposing 6-8 week old male mice to 0.005 ppm ozone for 2 h followed by 50 μg of intranasal LPS. We compared the immunomodulatory effects of single dose pre-treatment with CD61 blocking antibody (clone 2C9.G2), ATPase inhibitor BTB06584 against propranolol as the immune-stimulant and dexamethasone as the immune-suppressant in the ALI model. Ozone and LPS exposure induced lung neutrophil and eosinophil recruitment as measured by respective peroxidase (MPO and EPX) assays, systemic leukopenia, increased levels of lung vascular neutrophil regulatory chemokines such as CXCL5, SDF-1, CXCL13 and a decrease in immune-regulatory chemokines such as BAL IL-10 and CCL27. While CD61 blocking antibody and BTB06584 produced maximum increase in BAL leukocyte counts, protein content and BAL chemokines, these treatments induced moderate increase in lung MPO and EPX content. CD61 blocking antibody induced maximal BAL cell death, a markedly punctate distribution of NK1.1, CX3CR1, CD61. BTB06584 preserved BAL cell viability with cytosolic and membrane distribution of Gr1 and CX3CR1. Propranolol attenuated BAL protein, protected against BAL cell death, induced polarized distribution of NK1.1, CX3CR1 and CD61 but presented with high lung EPX. Dexamethasone induced sparse cell membrane distribution of CX3CR1 and CD61 on BAL cells and displayed very low lung MPO and EPX levels despite highest levels of BAL chemokines. Our study unravels ATPase inhibitor IF1 as a novel drug target for lung injury.

## Introduction

Lung inflammation is a common feature of many respiratory diseases like Acute Respiratory Distress Syndrome (ARDS) (1), asthma (2), Chronic Obstructive Pulmonary Disease (COPD) (3), and cystic fibrosis (4). It constitutes immune and neuro-humoral responses (5) from the vasculature and surrounding tissue in response to an infectious or other harmful stimulus. ARDS is a devastating complication of severe sepsis, from which patients have high mortality. Advances in treatment modalities including lung protective ventilation, prone positioning, use of neuromuscular blockade, and extracorporeal membrane oxygenation, have improved the outcome over recent decades; nevertheless, the mortality rate still remains high (6). Sepsis, ischemia reperfusion injury (as in stroke), hemodialysis, bacterial infections and multiple organ failure are marked by significant pulmonary neutrophil sequestration and concomitant neutropenia. This in turn, leads to immune suppression, which is another major cause of mortality (7, 8). Neutrophils are observed in lungs at post-mortem in ARDS patients and their numbers correlate with increasing severity of ARDS (9). The activated neutrophils mount a robust immune response; however, they also damage the blood-air barrier and cause lung injury (10, 11). The ultimate goal is to develop novel drug targets for lung inflammatory diseases without compromising the innate immune defense and the alveolar barrier.

Exposure to large concentrations (>1 ppm) of ozone for 6 h induces severe lung inflammation owing to its potent toxic effects (12, 13). We have recently shown that ambient ozone (from 0.005 to 0.5 ppm for 2 h) also induces instant cell death, marked by accumulation of vascular neutrophil and platelet aggregates albeit without inducing an increase in BAL protein or cell counts. We have also shown that low dose single ozone exposure of 0.05 ppm for 2 h impairs cell redox functions including the suppression and perturbation of innate immune response to bacterial lipopolysaccharide (14, 15). The dual burden of cell death and reduced immune capacity not only accumulates necrotic debris but also impairs the alveolar barrier and pathogen clearance (16, 17).

There are a few reports that point towards protection offered by propranolol in models of stroke, ozone-induced lung injury (5) and it’s atypical use in asthma patients refractory to long acting beta-adrenergic agonists (LABAs), largely owing to downregulation of adrenergic agonists (18). In a mouse stroke model lung neutrophil sequestration and concomitant lymphopenia in peripheral blood, thymus and spleen have been shown to solely respond to beta adrenergic blockade with propranolol (19). Glucocorticoids such as dexamethasone are one of the most powerful anti-inflammatory agents available clinically and work by inducing downregulation of the nuclear factor NFkB (20). Although this anti-inflammatory effect is attributed to inhibition of neutrophil recruitment it remains to be tested if glucocorticoids contribute to a delay in resolution of lung inflammation as the dynamics of these changes are not known. Moreover, glucocorticoids suppress the immune system, which is counter to the repair phase of inflammation. Thus, there is a need to develop novel immunomodulatory therapeutics which would not impair the innate immune response.

CD61 (Integrin β_3_) is widely expressed in lungs and is an integral regulator of cell adhesion and cell-surface signaling (21). CD61 induces cortical actin reorganization, thus stabilizing focal adhesions (22). We and others have shown that CD61 knock-out mice, blocking antibodies and peptides like RGD amplify vascular leak, neutrophil recruitment and worsen survival in acute lung injury and sepsis models (23, 24). Thus, CD61 is an important regulator of the alveolar barrier.

The F1F0 ATP synthase comprises a mitochondrial proton channel called F_o_, and a soluble catalytic F1 portion. The F1 portion comprises αβ heterodimers, which under favorable pH conditions (>6.5), synthesize ATP due to the proton motive force(25–27). Thus, ATP synthase is a chemo-mechanical protein (25). Pathophysiological stress (measured by a drop in mitochondrial potential) induced by ozone or LPS cause ectopic (surface) expression of the β subunit of ATP synthase (ATPβ), reversing the cellular energy flow (15, 28) which results in ATP hydrolysis and transport of protons against the gradient. We have shown *in vitro* that ATPβ is upregulated and surface-expressed during acute neutrophil polarization where it binds with exogenously administered angiostatin, endogenous CD61, prevents neutrophil pseudopod formation, stabilizes microtubules, inhibits mitochondrial activation and ROS production and induces neutrophil apoptosis (29). This suggests an essential role of ATP synthase in integrin β_3_ mediated neutrophil activation. Our *in vitro* observations were substantiated by *in vivo* studies where angiostatin administered subcutaneously (not intravenously) protected against LPS-induced lung vascular leak (30). Lung microangiography using state-of-the-art synchrotron dual K-edge subtraction imaging confirmed lung structure preservation by angiostatin treatment (31). The *in vitro* and *in vivo* data on the role of CD61 and angiostatin in lung injury, underscores the complex biology of ATP synthase in activated neutrophils.

Binding of angiostatin to surface-associated ATPβ has been reported by various research groups (32, 33). In the mitochondria, an additional protein, ‘‘inhibitor of F1’’ (IF1) is available to act as a ratchet ensuring that under conditions in which the electron motive force is diminished (such as relatively high pH below the membrane) the g chain of the F1 subunit cannot rotate in the clockwise direction and thus cannot needlessly consume ATP resources. Overexpression of mutant IF1in cell-lines or *in vivo* induces glycolytic enzymes and ROS production and protects against apoptosis and oxidative cell death (34, 35). A specific ATPase inhibitor, BTB06584 shows cardioprotection in ischemia reperfusion injury *via* IF1 (36). Because the contribution of ATPβ during lung inflammation is not well understood, we hypothesized that the ATPase inhibitor, BTB06584, regulates neutrophil recruitment and activation in a combined low-dose ozone and bacterial lipopolysaccharide (LPS) exposed or the “dual-hit” model of ALI.

Thus, the goals of our current study were to understand the inflammatory lung phenotypes in dual ozone and LPS exposed mice subjected to immune-modulation by CD61 blocking antibody, ATPase inhibitor BTB06584, propranolol or dexamethasone in murine ALI and to characterize the expression of the angiostatin and it’s binding proteins such as IF1 and CD61 in lung inflammation.

## Materials and methods

### Mice

The study design was approved by the University of Saskatchewan’s Animal Research Ethics Board and adhered to the Canadian Council on Animal Care guidelines for humane animal use. Six-eight week old male C57BL/6J (Stock No. 000664) mice were procured from Jackson Labs (CA, US).

### Reagents and chemicals

All chemicals utilized for the experiments were purchased from Sigma-Aldrich Chemicals (MO, US) unless otherwise indicated.

### Ozone exposures

For ozone exposures, mice were continuously exposed in an induction box for the desired times. These mice had free access to food and water while housed in the custom induction box. Ozone (0.005 ± 0.02 ppm) was generated, at 3 litres/minute, from ultra-high-purity air using a silent-arc discharge O_3_ calibrator cum generator (2B Technologies, CO, USA). Constant chamber air temperature (72 ± 3°F) and relative humidity (50 ± 15%) were maintained. Ozone concentrations were measured using a real-time ozone monitor (2B Technologies, CO, USA).

### Experiment design

**Figure 1:**
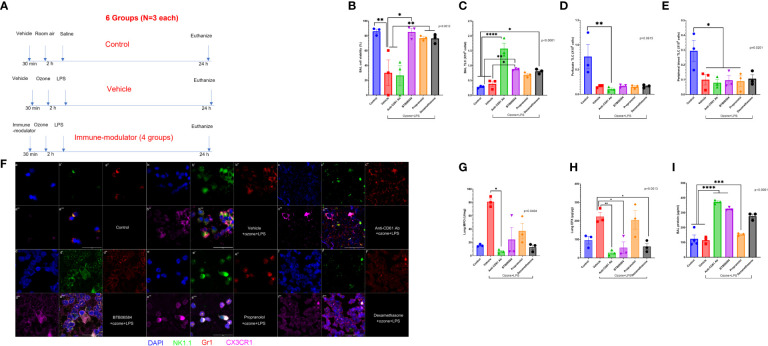
Effect of combined ozone and LPS exposure in mice: **(A)** Experiment design of the study showing treatments in 6 different groups with N=3 in each group. The first group represents control group with 0.001% DMSO administered intraperitoneally (IP) 30 minutes before housing mice in cage where they breathed room air for 2 h. After 2 h, mice were lightly anesthetized with IP ketamine/xylazine and instilled with 50 μl sterile saline. The second group represents treatment group with 0.001% DMSO administered intraperitoneally (IP) 30 minutes before housing mice in custom cage where they breathed 0.005 ppm ozone for 2 h. After 2 h, mice were lightly anesthetized with IP ketamine/xylazine and instilled with 50 μg/50μl bacterial lipopolysaccharide (LPS) from E. coli strain 055:B5. The third to sixth groups represent treatment group with immune-modulators (1 mg/kg anti-CD61 antibody (clone 2C9.G2), 1 mg/kg BTB06584, 1 mg/kg propranolol and 0.1 mg/kg dexamethasone) administered intraperitoneally (IP) 30 minutes before housing mice in custom cage where they breathed 0.005 ppm ozone for 2 h. After 2 h, mice were lightly anesthetized with IP ketamine/xylazine and instilled with 50 μg/50μl bacterial lipopolysaccharide (LPS) from E. coli strain 055:B5. All mice were euthanized at 24 h time-point to collect peripheral blood, vascular perfusate, bronchoalveolar lavage (BAL), right lung for biochemical and chemokine analysis and left lung fixed *in-situ* with 4% paraformaldeyde, for cryosectioning and H&E histology. **(B)** Percent BAL cell viability assessed with trypan blue exclusion, across the 6 groups, ANOVA p=0.0012, **(C)** BAL total leukocyte counts (TLC) expressed as 10^6^ cells, ANOVA p<0.0001, **(D)** Lung vascular perfusate TLC expressed as 10^6^ cells, Kruskal-Wallis p=0.0615, **(E)** Peripheral blood TLC expressed as 10^6^ cells, ANOVA p=0.0201. **(F)** BAL cell immune-fluorescent staining of control, and the 4 treatment groups, for nucleus with DAPI shown in blue (a, b, c, d, e, f), NK1.1 shown in green (a’, b’, c’, d’, e’, f’), Gr1 shown in red (a’’, b’’, c’’, d’’, e’’, f’’), CX3CR1 shown in fuschia (a’’’, b’’’, c’’’, d’’’, e’’’, f’’’). The merged panels are shown in the sub-parts a’’’’, b’’’’, c’’’’, d’’’’, e’’’’, f’’’’, scale = 50μm. **(G)** Lung myeloperoxidase (MPO) biochemical quantification across groups expressed as U/mg of lung tissue, Kruskal-Wallis p=0.0404, **(H)** Lung eosinophil peroxidase (EPX) ELISA based quantification across groups expressed as μg/μg of lung tissue, ANOVA p=0.0013, **(I)** BAL protein content across groups expressed as μg/ml, ANOVA p<0.0001. Significance level was set at p<0.05. * represents p<0.05, ** p<0.01, *** p<0.001, and **** p<0.0001.

Eighteen mice were randomized into six groups (one control, one vehicle+ozone+LPS and four immune-modulator pre-treated + ozone+LPS) with three mice per group. The control mice were pre-treated, 30 minutes before ozone exposure, as per respective IP doses, with vehicle (0.001% DMSO) and then returned to their cage to room air (RA) for 2 h. Mice were then anaesthetized intraperitoneally (IP) with ketamine (95 mg/kg) and xylazine (4.8 mg/kg) and instilled intranasally with 50 μl of sterile saline. These mice are represented as the control group (**Figure 1A**). For the treatment groups, mice were pre-treated 30 minutes before ozone exposure as per respective IP doses, with vehicle or the immune-modulator treatment groups which were as follows: 1 mg/kg anti-CD61 antibody (clone 2C9.G2), 1 mg/kg BTB06584, 1 mg/kg propranolol and 0.1 mg/kg dexamethasone. Mice from treatment groups were exposed to 0.005 ppm ozone for 2 h as explained above. Immediately after the 2 h ozone exposures, mice were anaesthetized intraperitoneally (IP) with ketamine (95 mg/kg) and xylazine (4.8 mg/kg) and instilled with 50 μg/50 μl LPS into the external nares.

At 24 h, the mice were anesthetized with IP ketamine (190 mg/kg) and xylazine (9.6 mg/kg) and prepared for further maneuvers. The experimental design is summarized in **Figure 1A**. Peripheral blood was collected in heparinized syringe by cardiac puncture. Mice were then tracheostomized, a custom non-collapsible polyethylene cannula was placed just before the end of tracheal bifurcation. Broncho-alveolar lavage (BAL) was collected with three consecutive washes, each with 0.5 ml PBS (phosphate buffered saline). The descending thoracic aorta was snipped at the mid-thoracic region. After clearing residual blood, lung vascular perfusate (LVP) was collected by perfusion through the right ventricle with PBS (0.25 ml X 2). The right lung lobes were ligated at the tracheo-bronchiolar hilus. The left lung was perfused with 0.5 ml freshly prepared 4% paraformaldehyde from a 20 cm water column to enable *in situ* fixation for 10 minutes and later cryo-embedded. Right lung was resected and flash frozen in liquid nitrogen and then stored at -80°C for further analysis. The collected samples are shown in the schematic in **Figure 1A**. BAL, peripheral blood and vascular perfusate were cytospun for immune-fluorescence with one slide stained for NK1.1, Gr1 and CX3CR1, and the other slide stained for ATPase IF1, Ki-67, CD61 and angiostatin. BAL supernatant was utilized for total leukocyte counts (TLC), protein, and 33-plex chemokine quantification. Right lung homogenates were prepared for MPO, EPX, quantifications. Vascular perfusate was quantified for TLC, 33-plex chemokine panel quantification. Peripheral blood samples were also analyzed for TLC, left lung cryosections were stained with hematoxylin and eosin (H&E) for histology.

### End-points

*a) Total (TLC) leukocyte counts:* Blood, BAL and lung vascular perfusate samples were centrifuged for 10 min at 3000 rpm. The supernatants were flash frozen and stored at -80°C until further analysis. The cells were reconstituted in PBS. TLC was performed by counting BAL, blood or lung perfusate cells on a hemocytometer. Trypan blue dye exclusion was utilized to quantify BAL cell viability under light microscopy. Blood TLC is presented as x10^6^ cells per mL. BAL and lung perfusate TLC are expressed in x10^3^ and x10^6^cells per collection, respectively. Acetic acid (2%) was added to lyse RBCs, in a 1:10 ratio for blood TLC and 1:2 ratio for lung vascular perfusate TLC. Cells (not more than 1X10^6^ per slide) were then centrifuged (Shandon cytospin, Thermoscientific, US) to prepare cyotspins.
*b) BAL cytospin NK1.1, Gr1, CX3CR1 staining:* BAL cytospin samples were prestained for the innate immune polymorphonuclear (Gr1), platelets (CX3CR1) or monocytic (NK1.1, CX3CR1) cell markers (2 µg mouse anti-NK1.1(clone PK136 IgG2ak), and 2 µg rat anti-Gr1 (clone RB68C5 IgG2bk) and 2 µg rabbit anti-CX3CR1 IgG (RRID: AB_467880), respectively). The respective secondary antibodies were incubated at 1μg/ml each of Alexa-488 conjugated anti-mouse IgG, Alexa-568 conjugated anti-rat IgG and Alexa-633 conjugated anti-rabbit IgG. Nuclei were stained with DAPI (4′,6-diamidino-2-phenylindole). Cytospin preparations were also stained with rat IgG2bk and mouse IgG2ak isotype control antibodies to validate the staining protocol. Images were acquired under a wide-field inverted Olympus microscope.
*c) BAL cytospin ATPase IF1, Ki-67, CD61 and angiostatin staining:* BAL cytospin samples were stained for the following markers: ATPase inhibitory factor (IF1), proliferation (Ki-67), focal adhesion and platelet marker (CD61) and anti-proliferation marker (angiostatin) (2 µg rabbit anti-IF1 IgG, and 2 µg rat anti-Ki-67 (clone SolA15 IgG2ak), 2 µg armenian hamster anti-CD61(2C9.G2 IgG1k) and 2 µg rabbit anti-angiostatin IgG, respectively). The respective secondary antibodies were incubated at 1μg/ml each of Alexa-305 conjugated anti-rabbit IgG, Alexa-488 conjugated anti-rat IgG, Alexa-568 conjugated anti-armenian hamster IgG and Alexa-633 conjugated anti-rabbit IgG. Cytospin preparations were also stained with rat IgG2ak and armenian hamster IgG1k isotype control antibodies to validate the staining protocol. Images were acquired under a wide-field inverted Olympus microscope.
*d) BAL protein analysis:* In order to quantify ozone induced edema, i.e., we measured total protein content in the collected BAL fluid. Supernatant fractions were analyzed for their total protein concentration using a standard colorimetric assay (Pierce 660 nm protein assay, Thermoscientific, IL, US).
*e) BAL and lung vascular perfusate chemokine analysis:* BAL and vascular perfusate supernatants were analyzed for chemokines using a 33-plex magnetic bead-based immunoassay (Bio-rad Laboratories Ltd., CA, US). The following chemokines were analyzed: CXCL13 (B-lymphocyte chemoattractant), CCL27 (IL-11 R-alpha-locus chemokine (ILC)), CXCL5 (epithelial-derived neutrophil-activating peptide 78 (ENA-78)), CCL-11 (eotaxin-1), eotaxin-2 (CCL-24), CX3CL1 (fractalkine), GM-CSF (CSF-2), CCL1, IFNγ (interferon gamma), IL-10 (interleukin-10), IL-16 (interleukin-16), IL-1β (interleukin-1 beta), IL-2 (interleukin-2), IL-4 (interleukin-4), IL-6 (interleukin-6), CXCL-10 (interferon gamma-induced protein 10 (IP-10)), CXCL11 (Interferon-gamma-inducible protein 9 (IP-9)), KC (keratinocyte chemoattractant), MCP-1 (monocyte chemoattractant protein-1), MCP-3 (monocyte chemoattractant protein-3), MCP-5 (monocyte chemoattractant protein-5), MDC (macrophage-derived chemokine (CCL22)), MIP-1α (macrophage inflammatory protein-1 alpha), MIP-1β (macrophage inflammatory protein-1 beta), MIP-2 (macrophage inflammatory protein-2), MIP-3α (macrophage inflammatory protein-3 alpha), MIP-3β (macrophage inflammatory protein-3 beta), RANTES (regulated on activation, normal T cell expressed and secreted (CCL5)), CXCL-16, CXCL-12/SDF-1alpha (stromal cell-derived factor 1), TARC (thymus and activation regulated chemokine (TARC)), TECK (Thymus-Expressed Chemokine (CCL25)) and TNFα (tumor necrosis factor alpha).
*f) Hematoxylin and eosin (H&E) histology:* H&E staining was performed on cryo-embedded lung sections for all groups.
*g) Lung Myeloperoxidase (MPO) 3,3′,5,5′-Tetramethylbenzidine (TMB) assay:* The right lung homogenates, in cetyl trimethyl ammonium chloride, were assayed, in duplicates, for MPO content which is based on direct peroxidase-catalyzed oxidation of TMB or indirect oxidation by hypochlorous acid and detected at 450 nm.
*h) Lung Eosinophil peroxidase (EPX) ELISA assay:* The right lung homogenates were assayed, in duplicates, *via* ELISA kit (Abbexa Ltd., UK, Catalog no. abx153959) which utilizes a proprietary capture antibody to detect the Val-252- Lys716 amino acid sequence of murine EPX.

### Statistical analysis

Results are expressed as mean ± SEM (N=3 per group). Effects of different treatments were analyzed by one way ANOVA or its nonparametric equivalent Kruskal-Wallis test followed by Tukey’s or Dunn’s multiple comparisons. A p value of <5% was considered significant. For multiplex chemokine analysis, one way ANOVA p values were adjusted for false discover rate by Benjamini-Hochberg correction. All analysis were done in GraphPad Prism 9 (Boston, MA, USA) Software, LLC.

## Results

### Bronchoalveolar lavage (BAL), perfusate and peripheral blood cell viability and total leukocyte counts (TLC)

Our previous studies report that ozone alone causes a substantial decrease in cell viability (40% viable cells in BAL, data not shown). The control group, i.e. vehicle+RA+saline, showed 86.21 ± 2.89% viable cells in BAL (**Figure 1B**). We observed a marked (2.83 fold) protection in BAL cell viability after BTB06584 treatment (85.30 ± 4.95% viable cells) compared to vehicle+ozone+LPS exposure (30.19 ± 17.40% viable cells, p<0.05) (**Figure 1B**). Anti-CD61 treatment was least protective with 26.56 ± 13.54% (p<0.01 vs control or BTB06584 group and p<0.05 vs propranolol or dexamethasone groups) dead cells followed by propranolol (77.00 ± 1.862% viable cells, p<0.05 vs vehicle+ozone+LPS group) and dexamethasone (76.53 ± 3.629% viable cells, p<0.05 vs vehicle+ozone+LPS group) (**Figure 1B**).

The total BAL leukocyte counts were not significantly different between control (0.28 ± 0.02X10^6^ cells) and vehicle+ozone+LPS (0.38 ± 0.09X10^6^ cells) group (**Figure 1C**). However, anti-CD61 antibody treatment led to maximum number of leukocytes in BAL (1.57 ± 0.18X10^6^ cells) which was 5.61 fold compared to control group (p<0.0001), 4.13 fold compared to vehicle+ozone+LPS group (p<0.0001), 1.81 fold compared to BTB06584 group (p<0.01), 2.28 fold compared to propranolol (p<0.001) and 1.94 fold compared to dexamethasone group (p<0.001) (**Figure 1C**). All immune-modulator treatment groups displayed leukopenia in the lung vascular perfusate (p=0.0615; p<0.05 for anti-CD61 antibody) (**Figure 1D**) as well as peripheral blood (p=0.0201; p<0.05 for vehicle+ozone+LPS, anti-CD61 antibody, BTB06584 and propranolol) (**Figure 1E**) compartment, when compared to control group.

### BAL, perfusate and peripheral blood innate immune cell immune-fluorescence

Nuclear morphology, Gr1, CX3CR1 and NK1.1 were used to characterize BAL, lung vascular perfusate and peripheral blood cells. Majority of BAL cells were mononuclear and Gr1 positive but a few were NK1.1, Gr1 and CX3CR1 triple positive in the vehicle+ozone+LPS group (**Figure 1F**-a to a’’’’; b to b’’’’). The vehicle+ozone+LPS treatment group also showed presence of lot of debris and CX3CR1 positive anuclear cells/fragments. We observed Gr1 positive polymorhonuclear cells, acellular CX3CR1 positive anuclear cells and NK1.1 positive mononuclear cells in the lung vascular perfusate (**Supplementary Figure 1a to a’**) and peripheral blood (**Supplementary Figure 2a to a’**) of vehicle treatment group when compared to the control group. Anti-CD61 antibody treatment led to accumulation of co-localized Gr1, NK1.1, and CX3CR1 triple positive cells in BAL which were distributed in a punctate fashion (**Figure 1F**-c to c’’’’) and a lot of debris in the lung vascular perfusate and peripheral blood (**Supplementary Figures 1a’’**, **2a’’**). BTB06584 treatment led to an increase in the number of Gr1, NK1.1 and CX3CR1 triple positive cells (**Figure 1F**-d to d’’’’) in BAL. These proteins were peripherally distributed in the BAL cells from BTB06584 treated group. The lung perfusate and peripheral blood showed predominantly triple NK1.1 and Gr1 double positive cells and CX3CR1 positive anuclear cells (**Supplementary Figures 1a’’’**, **2a’’’**). Isotype control staining provided evidence that the observed staining pattern in anuclear fragments was not non-specific. We observed 5-6 fold lower staining in the isotype controls for mouse IgG2ak, rat IgG2ak and rabbit IgG (**Supplementary Figure 3**). Propranolol led to slightly few Gr1, NK1.1 and CX3CR1 triple positive cells (**Figure 1F**-e to e’’’’) in all compartments (**Supplementary Figures 1a’’’’**, **2a’’’’**). BAL cells in the propranolol group displayed polarized distribution of these protein markers. Dexamethasone treatment led to accumulation of Gr1 positive cells with peripheral distribution of Gr1 (**Figure 1F**-f to f’’’’) in BAL, compromised cells as well as cells with intracellular distribution of Gr1 and CX3CR1 in the vascular perfusate and peripheral blood (**Supplementary Figures 1a’’’’’**, **2a’’’’’**).

### Lung myeloperoxidase (MPO) and eosinophil peroxidase (EPX)

To ascertain the number of neutrophils or eosinophils left after BAL, MPO and EPX were quantified in lung homogenates. To our surprise, we observed highest MPO in vehicle+ozone+LPS (81.00 ± 4.43 U/mg, p<0.05) but significance was only achieved against anti-CD61 antibody (6.61 ± 1.67 U/mg) treated group (**Figure 1G**).

Lung EPX was highest in the vehicle+ozone+LPS (223.2 ± 22.81 μg/μg) group followed by propranolol (205.3 ± 50.75 μg/μg) when compared to anti-CD61 antibody (28.35 ± 8.43 μg/μg, p<0.01), BTB06584 (55.50 ± 31.21 μg/μg, p<0.05) and dexamethasone group (61.39 ± 17.61 μg/μg, p<0.05) (**Figure 1H**).

### BAL protein

BAL protein was different across the control and treatment groups (p<0.0001) (**Figure 1I**). BAL protein was not significantly different between control and vehicle+ozone+LPS group. Both anti-CD61 antibody (373.0 ± 7.65 μg/ml) and BTB06584 (325.3 ± 6.15 μg/ml) treatment groups had high BAL protein compared to the control (123.8 ± 26.69 μg/ml), vehicle+ozone+LPS (114.9 ± 13.57 μg/ml) and propranolol (153.5 ± 5.29 μg/ml) groups (p<0.0001) (**Figure 1I**). Dexamethasone treatment led to higher BAL protein (277.0 ± 13.90 μg/ml) compared to both control and vehicle+ozone+LPS groups (p<0.001) as well as the propranolol (p<0.01) group (**Figure 1I**). BAL protein in the anti-CD61 antibody group was higher than the dexamethasone group (p<0.05) (**Figure 1I**).

### BAL and perfusate chemokines

**Figure 2:**
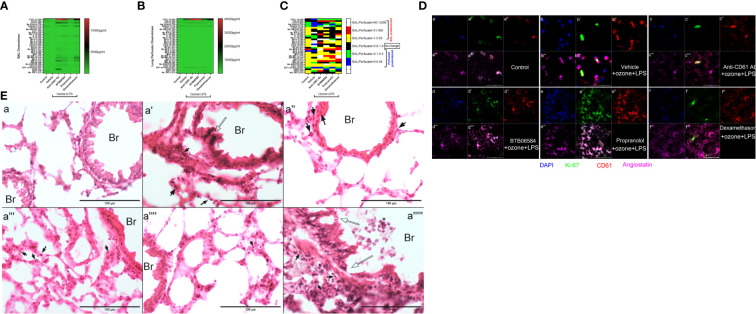
Chemokine, molecular and histology. **(A)** Bronchoalveolar lavage (BAL) fluid 33-plex chemokine analysis expressed as pg/ml in heat map, **(B)** Lung vascular perfusate 33-plex chemokine analysis expressed as pg/ml in heat map and **(C)** BAL/vascular perfusate ratio of 33 chemokines across the six groups: control, and 5 treatment groups namely vehicle, CD61 blocking (anti-CD61) antibody, BTB06584, propranolol and dexamethasone treatments. For magnetic bead-based immunoassay (Bio-rad Laboratories Ltd., CA, US), the following chemokines were analyzed: CXCL13 (B-lymphocyte chemoattractant), CCL27 (IL-11 R-alpha-locus chemokine (ILC)), CXCL5 (epithelial-derived neutrophil-activating peptide 78 (ENA-78)), CCL-11 (eotaxin-1), eotaxin-2 (CCL-24), CX3CL1 (fractalkine), GM-CSF (CSF-2), CCL1, IFNγ (interferon gamma), IL-10 (interleukin-10), IL-16 (interleukin-16), IL-1β (interleukin-1 beta), IL-2 (interleukin-2), IL-4 (interleukin-4), IL-6 (interleukin-6), CXCL-10 (interferon gamma-induced protein 10 (IP-10)), CXCL11 (Interferon-gamma-inducible protein 9 (IP-9)), KC (keratinocyte chemoattractant), MCP-1 (monocyte chemoattractant protein-1), MCP-3 (monocyte chemoattractant protein-3), MCP-5 (monocyte chemoattractant protein-5), MDC (macrophage-derived chemokine (CCL22)), MIP-1α (macrophage inflammatory protein-1 alpha), MIP-1β (macrophage inflammatory protein-1 beta), MIP-2 (macrophage inflammatory protein-2), MIP-3α (macrophage inflammatory protein-3 alpha), MIP-3β (macrophage inflammatory protein-3 beta), RANTES (regulated on activation, normal T cell expressed and secreted (CCL5)), CXCL-16, CXCL-12/SDF-1alpha (stromal cell-derived factor 1), TARC (thymus and activation regulated chemokine (TARC)), TECK (Thymus-Expressed Chemokine (CCL25)) and TNFα (tumor necrosis factor alpha) **(D)** BAL cell immune-fluorescent staining of control, and 4 treatment groups, for ATPase inhibitory factor 1 (IF1) shown in blue (a, b, c, d, e, f), Ki-67 shown in green (a’, b’, c’, d’, e’, f’), CD61 shown in red (a’’, b’’, c’’, d’’, e’’, f’’), Angiostatin shown in fuschia (a’’’, b’’’, c’’’, d’’’, e’’’, f’’’). The merged panels are shown in the sub-parts a’’’’, b’’’’, c’’’’, d’’’’, e’’’’, f’’’’, scale = 50μm. **(E)** Lung cryosection H&E across groups i.e. control (a), and 5 treatment groups namely vehicle (a’), CD61 blocking (anti-CD61) antibody (a’’), BTB06584 (a’’’), propranolol (a’’’’) and dexamethasone (a’’’’’) treatments. Scale bar = 100 μm, Br=bronchus, open arrows indicate bronchiolar epithelial damage, closed arrows indicate polymorphonuclear cells majority of which are eosinophilic in appearance, * represents clusters of polymorphonuclear cells. Note the dual color i.e. dark purple and eosinophilic presentation of lungs after ozone and LPS treatment in images from a’ to a’’’’’.

Out of the 33 chemokines analyzed in BAL, only CXCL5 showed a slight increase in the BAL compartment after vehicle+ozone+LPS (100.77 ± 47.66 pg/ml) treatment compared to control (146.63 ± 35.85 pg/ml) group, but did not achieve statistical significance (**Figure 2A**). Instead, we observed significant decrease in BAL CXCL13 (2.5 fold, p<0.0001), CCL27 (2.9 fold, p<0.0001), eotaxin-2 (6.7 fold, p<0.0001), IL-10 (9.2 fold, p<0.01) and TECK (3 fold, p<0.01) after vehicle+ozone+LPS treatment when compared to control group (**Figure 2A**). In the perfusate compartment, levels of not only CXCL5 (2.2 fold, p<0.0001), but also CXCL13 (8.7 fold, p<0.0001) and SDF-1α (4.8 fold, p<0.0001) increased in the vehicle+ozone+LPS group when compared to the control group (**Figure 2B**). Levels of CCL27 (3.2 fold, p<0.01) and eotaxin-2 (1.4 fold, p<0.05) was found to be lower in the perfusate of vehicle+ozone+LPS group compared to control group (**Figures 2B, C**).

Anti-CD61 antibody treatment induced maximum increase in BAL levels of CXCL13 (14.6 fold, p<0.0001), CXCL5 (29.5 fold, p<0.0001), KC (15.1 fold, p<0.05), MCP-1 (11.7 fold, p<0.0001), MCP-3 (36.9 fold, p<0.05), MDC (7.7 fold, p<0.01), MIP-1a (83.8 fold, p<0.0001), MIP-1b (44.0 fold, p<0.0001), MIP-2 (14.1 fold, p<0.0001), MIP-3a (141.5 fold, p<0.0001), RANTES (14.7 fold, p<0.0001), TARC (58.2 fold, p<0.0001), and moderate yet significantly high levels of BAL IL-16 (7.1 fold, p<0.05) vs vehicle+ozone+LPS group (**Figure 2A**). The levels of perfusate CXCL13 (34.2 fold, p<0.0001), CXCL5 (2.6 fold, p<0.0001) and SDF-1α (6.7 fold, p<0.0001) were also highest following anti-CD61 antibody treatment when compared to control group (**Figure 2B**).

BTB06584 induced maximal increase in the BAL levels of IL-16 (11.8 fold, p<0.0001) and RANTES (20.9 fold, p<0.0001) (**Figures 2A, C**). The BAL levels of CXCL13 (12.6 fold, p<0.0001), CXCL5 (22.1 fold, p<0.0001), eotaxin-2 (1.4 fold, p<0.001), IL-6 (8.4 fold, p=0.064), MIP-1a (51.0 fold, p<0.001), MIP-1b (28.0 fold, p<0.0001), MIP-2 (18.8 fold, p<0.0001) and MIP-3a (62.2 fold, p<0.0001) were also higher in the BTB06584 treated group compared to control group (**Figures 2A, C**). The levels of perfusate CXCL13 (37.0 fold, p<0.0001), CXCL5 (2.0 fold, p<0.0001) and SDF-1α (3.7 fold, p<0.0001) were also high following BTB06584 treatment when compared to control group (**Figures 2B, C**).

Propranolol treatment induced increase in levels of BAL CXCL13 (1.9 fold, p<0.0001), CXCL5 (7.6 fold, p<0.0001), MIP-1b (11.6 fold, p<0.01), TARC (7.0 fold, p<0.01) compared to control group (**Figures 2A, C**). The levels of perfusate CXCL13 (9.9 fold, p<0.0001), CXCL5 (1.9 fold, p<0.0001), SDF-1α (5.4 fold, p<0.0001) and TECK (9.5 fold, p<0.0001) were also high following propranolol treatment when compared to control group (**Figures 2B, C**).

Dexamethasone treatment induced maximal increase in levels of BAL eotaxin-2 (2.1 fold, p<0.0001), IL-6 (13.8 fold, p<0.0001), MIP-1a (91.7 fold, p<0.0001), MIP-1b (83.1 fold, p<0.0001), and MIP-2 (29.1 fold, p<0.0001), CXCL16 (2.3 fold, p<0.01) (**Figures 2A, C**). Levels of BAL CXCL13 (8.9 fold, p<0.0001), CXCL5 (19.5 fold, p<0.0001), IL-16 (10.8 fold, p<0.0001), MIP-3a (69.9 fold, p<0.0001) and RANTES (12.2 fold), were also high after dexamethasone treatment when compared to the control group (**Figures 2A, C**). The levels of perfusate CXCL13 (16.0 fold, p<0.0001) and SDF-1α (2.7 fold, p<0.05) were also high following dexamethasone treatment when compared to control group (**Figures 2B, C**). All the immune modulator treatments led to a decrease in BAL as well as perfusate CCL-27 and BAL IL-10 levels (**Figures 2A, B**).

### BAL ATPIF1, Ki-67, CD61 and angiostatin immune-fluorescence

In order to look at the expression of focal adhesions, magakaryocyte and platelet expressing CD61, IF1, anti-proliferation marker angiostatin and the proliferation factor Ki-67, we sought to image these proteins in BAL cytospins. We observed higher IF1 expression in vehicle+ozone+LPS treated BAL cells compared to control group (**Figure 2D**-a). Although we could not ascertain their distribution in the nuclear compartment, the distribution pattern of IF1 suggested its nuclear expression. Intracellular CD61 and angiostatin expression was also diffuse in BAL cells in control (**Figure 2D**-a’’, a’’’) and vehicle+ozone+LPS treated groups (**Figure 2D**-b’’, b’’’). There was a predominant Ki-67 expression in vehicle+ozone+LPS group indicating the stimulation of cell proliferation in response to these exposures (**Figure 2D**-b’). Numerous small CD61 and angiostatin positive entities were also observed in the BAL of vehicle+ozone+LPS treated cytospins (**Figure 2D**-b’’, b’’’). The anti-CD61 antibody treated BAL cells displayed moderate staining for IF1, CD61, angiostatin and Ki-67 (**Figure 2D**-c, c’, c’’, c’’’). BTB06584 treatment induced acellular CD61 (**Figure 2D**-d’’) and angiostatin (**Figure 2D**-d’’’) positive punctate vesicles that were also positive for IF1 (**Figure 2D**-d). Propranolol, on the other hand, induced diffuse distribution of cells positive for IF1 (**Figure 2D**-e), CD61 (**Figure 2D**-e’’), angiostatin (**Figure 2D**-e’’’) and Ki-67 (**Figure 2D**-e’). Dexamethasone treatment induced peripheral distribution of CD61 (**Figure 2D**-f’’) and angiostatin (**Figure 2D**-f’’’) in BAL cells. A small fraction of cells were positive for Ki-67 (**Figure 2D**-f’) and IF1 (**Figure 2D**-f) as well.

### Lung H&E staining

Lung cryosections revealed wide-spread alveolar and bronchiolar damage, hemorrhage, vascular leukocyte recruitment in vehicle (**Figure 2E**-a’), CD61 blocking antibody (**Figure 2E**-a’’) and dexamethasone treatment groups (**Figure 2E**-a’’’’’). Open arrows indicate bronchiolar epithelial damage, closed arrows indicate polymorphonuclear cells majority of which are eosinophilic in appearance, * represents clusters of polymorphonuclear cells noted especially in dexamethasone treated lung section (**Figure 2E**-a’’’’’). Note the dual color i.e. dark purple and eosinophilic presentation of lungs after ozone and LPS treatment in images from **Figure 2E**-a’ to a’’’’’.

## Discussion

We have recently studied the effects of ozone alone in a time (up to 24 h) and dose (from 0.005 to 0.5 ppm) dependent manner (14). In addition, we have also studied the effects of LPS alone (30), and ozone+LPS (15) up to 72 h. We have shown that low dose ozone (0.05 ppm) does not induce increase in BAL total protein content. One of the reasons for lack of change in BAL protein could be due to protein degradation and wide-spread cell death as we do observe the cardinal signs of phlogistic cell death, extracellular DNA in BAL, vascular perfusate, and peripheral blood. We observed neutrophils, neutrophil and eosinophil chemokines in the vasculature, MPO in the lung tissue and large monocytic CD11b bright cells in BAL. The main reason for choosing the current ozone and LPS model at 24 h is based on these observations where lung inflammation is suppressed and delayed by 0.05 ppm ozone and 50 μg LPS; BAL protein peaked at 36 h, BAL cell counts peaked at 72 h unlike the BAL counts seen in ozone alone (peak at 6 h) or LPS alone (peak at 9 h). As very low doses of ozone (0.005 ppm) also compromise the alveolar barrier, induce BAL cell death and LPS is a strong neutrophil and innate immune response inducer, we wanted to assess the effects of novel immune interventions on innate immune response induced by the combined exposure to ozone and LPS.

The current model utilizes 0.005 ppm ozone followed by LPS exposure resulting in a suppressed immune response while inducing lung MPO and EPX release which are direct indicators of neutrophil and eosinophil accumulation and degranulation (37). We further investigated the effects of immune stimulator propranolol and immune-suppressant dexamethasone which were compared to ATPase inhibitor, BTB06584 and CD61 blocking antibody. We have compiled our results in a comparative table (**Table 1**). We found that BTB06584 protects against cell death, induces robust lung neutrophil recruitment but does not induce massive eosinophil or neutrophil peroxidase release when compared to vehicle treated group.

**Table 1:** Comparison of the main parameters, namely bronchoalveolar lavage (BAL) total leukocyte counts (TLC), BAL viability, BAL protein, BAL chemokines, lung vascular perfusate TLC, perfusate chemokines, peripheral blood TLC, lung myeloperoxidase (MPO) and lung eosinophil peroxidase (EPX).

Parameter	Control	Vehicle	Anti-CD61 Ab	BTB06584	Propranolol	Dexamethasone
BAL TLC	+	+	+++++	+++	+++	+++
BAL Viability	++++++	+	+	++++++	++++	++++
BAL Protein	+	+	++++++	++++++	+	++
BAL Chemokines	CCL-27, IL-10	High but not significant: CXCL5	Highest ↑: CXCL13, CXCL5, KC, MCP-1, MCP-3, MDC, MIP-1a, MIP-1b, MIP-2, MIP-3a, RANTES, TARC Moderate ↑: IL-16	Highest ↑: IL-16, RANTES Moderate: CXCL13, CXCL5, eotaxin-2, IL-6, MIP-1a, MIP-1b, MIP-2, MIP-3a	Moderate ↑: CXCL13, CXCL5, MIP-1b, TARC	Highest ↑: eotaxin-2, IL-6, MIP-1a, MIP-1b, MIP-2, CXCL16 Moderate ↑: CXCL13, CXCL5, IL-16, MIP-3a, RANTES
Perfusate TLC	++++++	+	+	+	+	+
Perfusate Chemokines		Moderate ↑: CXCL5, SDF-1α, CXCL13	Highest ↑: CXCL5, SDF-1α, CXCL13	Highest ↑: CXCL13 Moderate ↑: CXCL5, SDF-1α	Moderate ↑: CXCL5, SDF-1α, CXCL13, TECK	Moderate ↑: SDF-1α, CXCL13
Peripheral Blood TLC	++++++	+	+	+	+	+
Lung MPO	+	++++++	+	++	++	+
Lung EPX	+	++++++	+	++	++++	+

The symbols + to ++++ stand for the graded magnitude of the observed parameter in each group with + being the least and ++++ being the maximum. The symbol ↑ stands for an increase in the parameter described.

Nuclear morphology, Gr1, CX3CR1 and NK1.1 fluorescence were used to characterize the BAL, lung vascular perfusate and peripheral blood cells. BAL cell counts and immune-fluorescent images showed large numbers of Gr1 and CX3CR1 double positive polymorphonuclear cells in the CD61 blocking antibody, BTB06584, propranolol and dexamethasone treatment groups compared to control or vehicle (ozone+LPS) treatment groups. Importantly, we observed debris, anuclear CX3CR1 and CD61 positive cells which could likely be platelets (38) or cell fragments called as microparticles (39). BAL cells from control, BTB06584, propranolol and dexamethasone treatment groups showed protection against cell death rather than signs of proliferation as visualized in the reduced Ki-67 staining in the immune-modulator treated groups compared to vehicle treatment group. Vehicle as well as immune-modulator treated groups showed leukopenia compared to control group. Only the CD61 blocking antibody, BTB06584 and dexamethasone treated mice showed high BAL protein compared to control, vehicle and propranolol treatment groups. This increase in BAL protein could be a result of either enhanced vascular permeability or higher amount of protein secreted in the BAL, which is a contentious hypothesis to be tested in carefully planned experiments. The vascular, and not the BAL, compartment of vehicle treated group showed predominance of neutrophil chemokine CXCL5, SDF-1α and the B cell chemokine, CXCL13, as indicated by the BAL to vascular perfusate chemokine ratios. These results are in agreement with our earlier study where the chemokine signature was similar after a single 0.05 ppm ozone+LPS exposure (15). All the treatment groups showed decrease in levels of regulatory BAL chemokines, namely IL-10 and CCL27. CD61 blocking antibody, BTB06584 and dexamethasone treatments induced significant increase in BAL chemokines namely RANTES, CXCL5, MIP-1a, MIP-1b, MIP-2, MIP-3a, IL-16, CXCL13, MCP-1, MCP-3, with KC, MDC and TARC being specific to CD61 blocking antibody treatment, eotaxin-2, IL-6 being specific to BTB06584 and IL-6, CXCL16 specific to dexamethasone treatment. Propranolol treatment led to increase in levels of BAL CXCL5, MIP-1a, CXCL13 and TARC. IL-16, KC, CXCL5, CXCL12 (i.e. SDF-1α), MIP-2 are important neutrophil chemokines (40–43). CXCL13 is a lymphoid expressed chemokine that induces B cell migration and is also a regulator of neutrophil migration (44). Other MIP isoforms are chemotactic for monocytes and lymphocytes. Eotaxin-2, RANTES and MCP-1 are known to mediate bronchial hyperresponsiveness, recruitment of mononuclear cells, eosinophil, platelet activation and allergic inflammation (45). Macrophage derived TARC and MDC are chemotactic not only for Th2 cells but also epithelial cells (46). The control, CD61 blocking antibody and dexamethasone treatment groups showed near baseline levels of MPO and EPX. Despite the non-significant increase in BAL leukocytes, the lungs of vehicle treated group had the maximum amount of myeloperoxidase (MPO). The lungs of vehicle treated group also had the maximum amount of eosinophil peroxidase (EPX) which was moderately increased in propranolol and BTB06584 treatment groups. Interestingly, we observed numerous polymorphonuclear cells in lung sections, majority of which were eosinophilic in appearance. Dexamethasone treated lung showed clusters of eosinophilic cells. The data from H&E histology and peroxidase assays suggests that the polymorphonuclear neutrophils and eosinophils are more adherent after ozone and LPS treatment, suggesting their activation.

Taken together, our results indicate that both neutrophils and eosinophils orchestrate lung inflammation after single exposure to just 0.005 ppm ozone and 50 μg of intranasal LPS. The lungs present with widespread alveolar and bronchiolar damage, which was markedly exaggerated after pre-treatment with single IP dose of CD61 blocking antibody. The immunomodulators tested in our study induced significant protein and chemokine content in BAL as well as perfusate but BTB06584, propranolol and dexamethasone protected against excessive cell death, which might afford some degree of protection from ozone’s toxic effects. Although our study was designed to analyze the effects of different treatments on acute lung inflammation parameters at 24 h time-point, it would be interesting to follow these parameters at later time-points and assess the late phase of inflammation and resolution.

## Data availability statement

The raw data supporting the conclusions of this article will be made available by the authors, without undue reservation.

## Ethics statement

The animal study was reviewed and approved by University Animal Care Committee (UACC).

## Author contributions

GA secured funding (NSERC-DG), designed, executed the study and analyzed the results. PS performed downstream assays on lung homogenates. All authors contributed to the article and approved the submitted version.

## Supplementary material

Supplementary Figure 1 Lung vascular perfusate immune-fluorescent staining of control (a), 4 treatment groups i.e. vehicle (a’), anti-CD61 antibody (a’’), BTB06584 (a’’’), propranolol (a’’’’) and dexamethasone (a’’’’’) with merged panels showing nucleus stained with DAPI in blue, NK1.1 shown in green, Gr1 shown in red, CX3CR1 shown in fuschia, scale = 50μm.

Supplementary Figure 2 Peripheral blood immune-fluorescent staining of control (a), 4 treatment groups i.e. vehicle (a’), anti-CD61 antibody (a’’), BTB06584 (a’’’), propranolol (a’’’’) and dexamethasone (a’’’’’) with merged panels showing nucleus stained with DAPI in blue, NK1.1 shown in green, Gr1 shown in red, CX3CR1 shown in fuschia, scale = 50μm.

Supplementary Figure 3 Representative isotype immune-fluorescent staining of ozone and LPS treated peripheral blood with nucleus stained with DAPI in blue, mouse IgG2ak shown in green, rat IgG2ak shown in red, rabbit IgG shown in fuschia and merged panel, scale = 50μm.
