# Supplementary figures and images for: Modulation of low-dose ozone and LPS exposed acute mouse lung inflammation by IF1 mediated ATP hydrolysis inhibitor, BTB06584
Source: Front Immunol. 2023 Mar 13;14:1126574. doi: 10.3389/fimmu.2023.1126574 (PMC10040673; doi:10.3389/fimmu.2023.1126574)

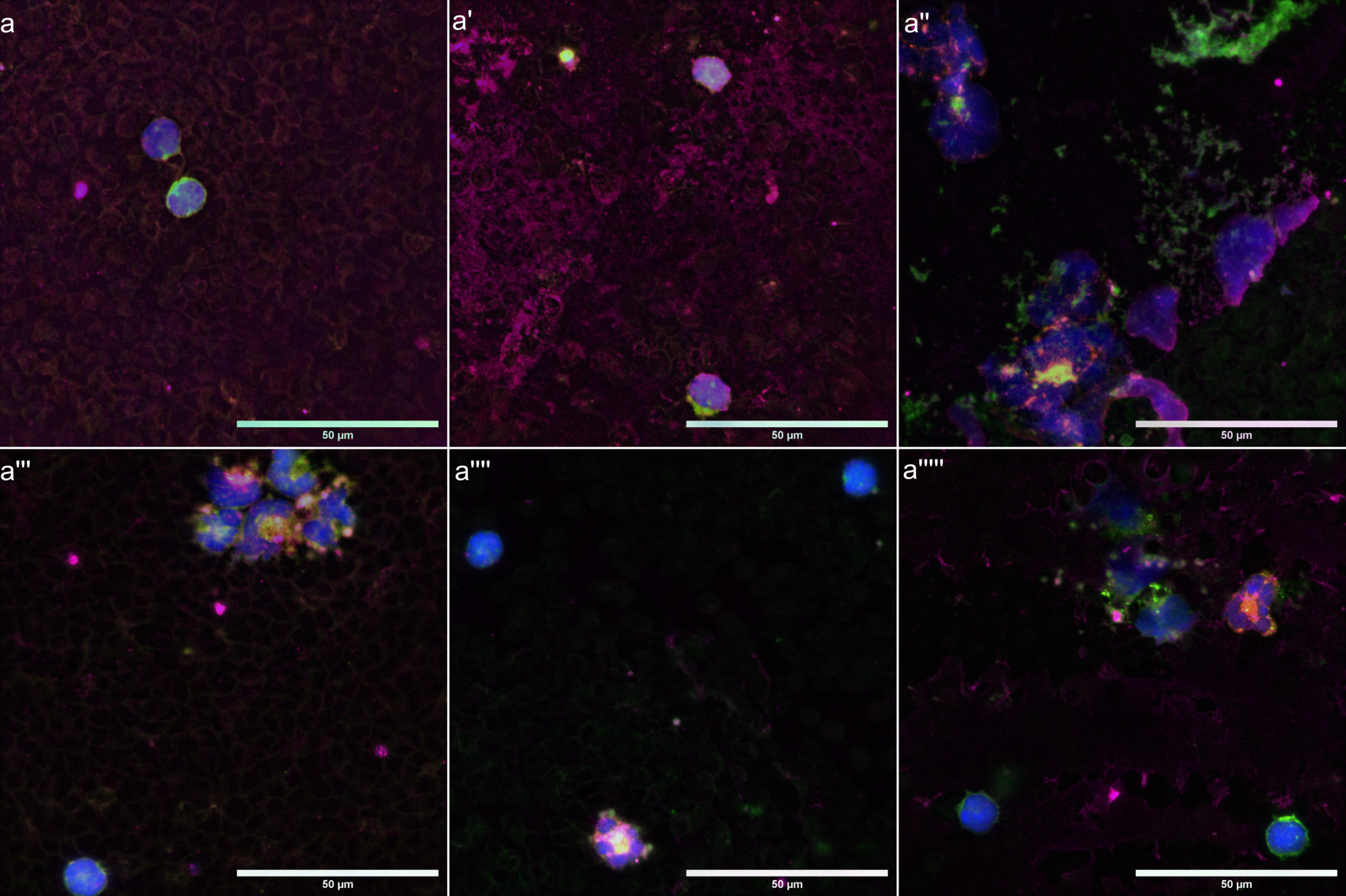

Supplement: Supplementary Figure 1 — Lung vascular perfusate immune-fluorescent staining of control (a), 4 treatment groups i.e. vehicle (a’), anti-CD61 antibody (a’’), BTB06584 (a’’’), propranolol (a’’’’) and dexamethasone (a’’’’’) with merged panels showing nucleus stained with DAPI in blue, NK1.1 shown in green, Gr1 shown in red, CX3CR1 shown in fuschia, scale = 50μm. [file Image_1.tif]

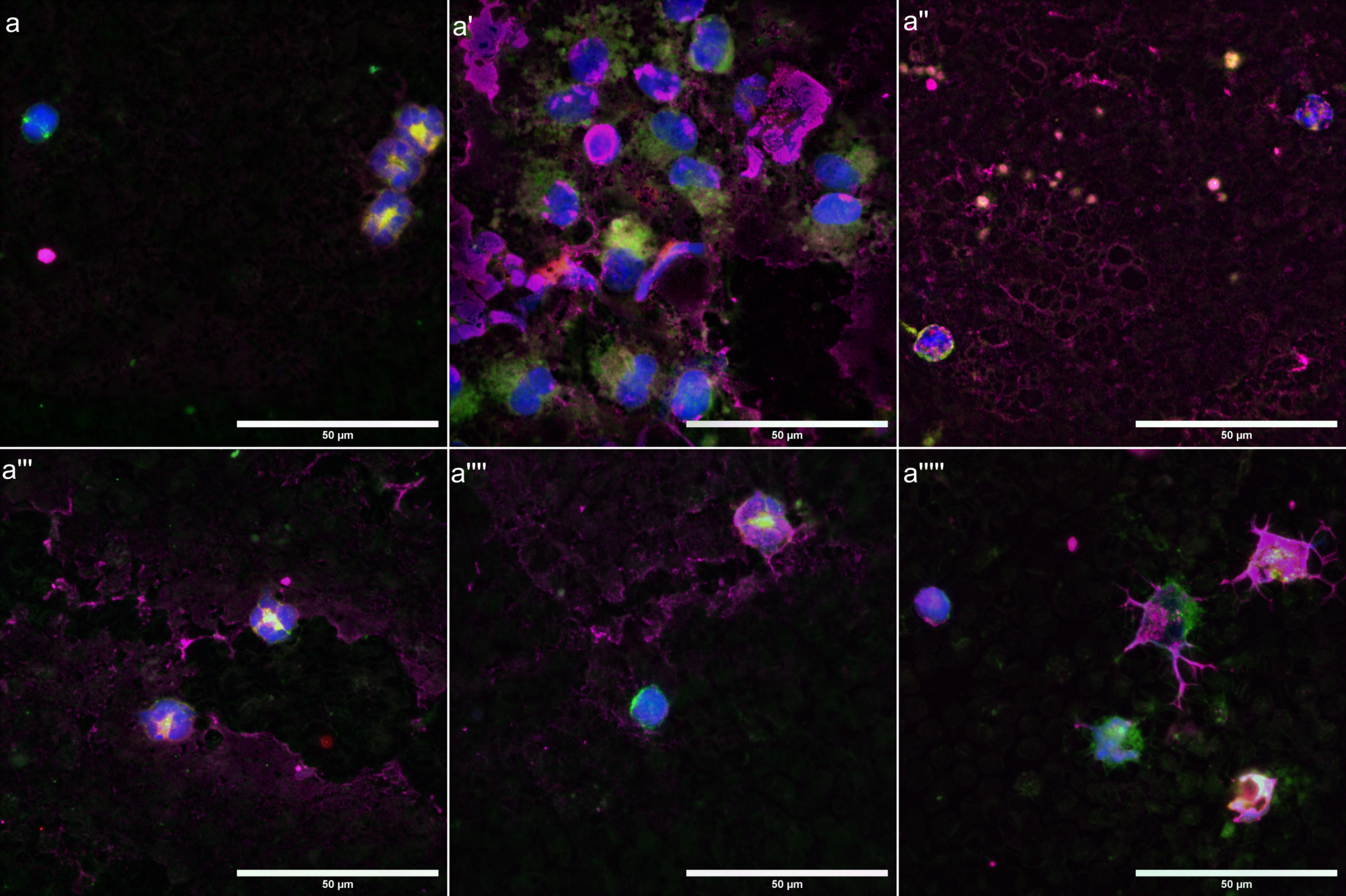

Supplement: Supplementary Figure 2 — Peripheral blood immune-fluorescent staining of control (a), 4 treatment groups i.e. vehicle (a’), anti-CD61 antibody (a’’), BTB06584 (a’’’), propranolol (a’’’’) and dexamethasone (a’’’’’) with merged panels showing nucleus stained with DAPI in blue, NK1.1 shown in green, Gr1 shown in red, CX3CR1 shown in fuschia, scale = 50μm. [file Image_2.tif]

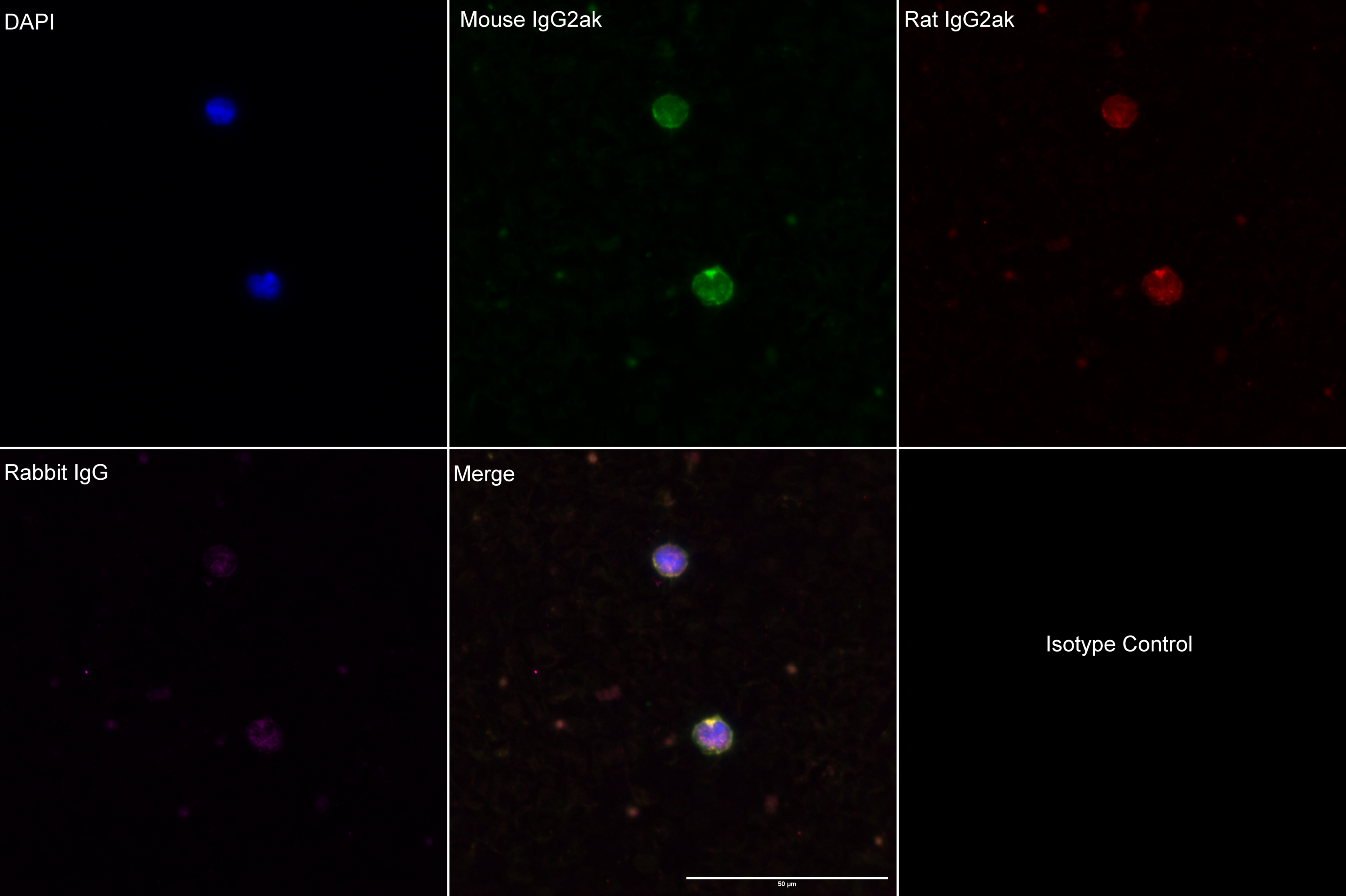

Supplement: Supplementary Figure 3 — Representative isotype immune-fluorescent staining of ozone and LPS treated peripheral blood with nucleus stained with DAPI in blue, mouse IgG2ak shown in green, rat IgG2ak shown in red, rabbit IgG shown in fuschia and merged panel, scale = 50μm. [file Image_3.tif]
